# Tsetse fly microbiota: form and function

**DOI:** 10.3389/fcimb.2013.00069

**Published:** 2013-10-29

**Authors:** Jingwen Wang, Brian L. Weiss, Serap Aksoy

**Affiliations:** Department of Epidemiology of Microbial Diseases, Yale School of Public HealthNew Haven, CT, USA

**Keywords:** tsetse fly, symbiont, *wigglesworthia*, *Sodalis*, *wolbachia*

## Abstract

Tsetse flies are the primary vectors of African trypanosomes, which cause Human and Animal African trypanosomiasis in 36 countries in sub-Saharan Africa. These flies have also established symbiotic associations with bacterial and viral microorganisms. Laboratory-reared tsetse flies harbor up to four vertically transmitted organisms—obligate *Wigglesworthia*, commensal *Sodalis*, parasitic *Wolbachia* and Salivary Gland Hypertrophy Virus (SGHV). Field-captured tsetse can harbor these symbionts as well as environmentally acquired commensal bacteria. This microbial community influences several aspects of tsetse's physiology, including nutrition, fecundity and vector competence. This review provides a detailed description of tsetse's microbiome, and describes the physiology underlying host-microbe, and microbe-microbe, interactions that occur in this fly.

## Introduction

Tsetse flies (*Glossina* sp.) serve as hosts to numerous microorganisms. This insect is the primary vector of *Trypanosoma brucei* parasites that cause a chronic wasting disease in humans (human African trypanosomiasis, or HAT) and domesticated animals (animal African trypanosomiasis, or AAT) in 36 countries throughout sub-Saharan Africa. Both HAT and AAT are pernicious diseases that inflict untold hardships upon their mammalian hosts. In fact, African trypanosomes are regarded as one of the greatest constraints to Africa's economic development (Simarro et al., 2008; Welburn and Maudlin, 2012).

Tsetse also has a relationship with multiple bacterial species and at least one virus. Tsetse's bacterial partners can include 3 maternally-transmitted endosymbionts as well as a taxonomically diverse collection of commensals that the fly acquires from it's environment. Additionally, many tsetse flies also harbor a salivary gland-associated DNA virus. These microbes are intimately associated with many important aspects of their host's biology. Thus, increasing our fundamental knowledge regarding how tsetse interacts with it's microbiota will allow us to develop novel and innovative control strategies aimed at reducing tsetse populations and/or tsetse vector competence.

## Exploiting tsetse's unusual reproductive physiology for population control

Tsetse flies lead a relatively sterile existence when compared to the vast majority of other insects. Adult males and females feed exclusively on sterile vertebrate blood. Also, unlike other oviparous insects, female tsetse produce only one egg per gonotrophic cycle (Tobe, 1978). Tsetse offspring develop in their mother's uterus. 3rd instar larvae are deposited and immediately pupate. Adult flies emerge 30 days later. This mode of reproduction results in deposition of only 8–10 progeny per female over her lifespan.

During larvagenesis, tsetse progeny receive nourishment via highly modified maternal accessory gland (referred to as the milk gland) secretions that contain proteins, lipids and amino acids (Attardo et al., 2008, 2012; Benoit et al., 2012; Wang and Aksoy, 2012). Several milk associated protein-encoding genes, including *milk gland proteins (mgp) 1–10*, *transferrin* and *acid sphingomyelinase 1*, are differentially expressed during pregnancy in the milk gland organ. All 10 milk proteins are up-regulated during lactation to support larval growth, and then down-regulated drastically following parturition. Interference with the expression of milk proteins through a double-stranded RNA interference approach has negative effects on fecundity by extending larvagenesis and/or inducing premature abortions (Attardo et al., 2010; Yang et al., 2010; S. Aksoy and J. Benoit, personal communication). Thus, compounds that interfere with milk production could be used as novel vector control tools to reduce vector populations. Understanding the mechanism(s) that regulates the coordinated transcription of milk proteins may lead to the discovery of key regulatory factors that could be exploited to reduce fecundity.

## The biology of tsetse symbiosis

**Figure 1:**
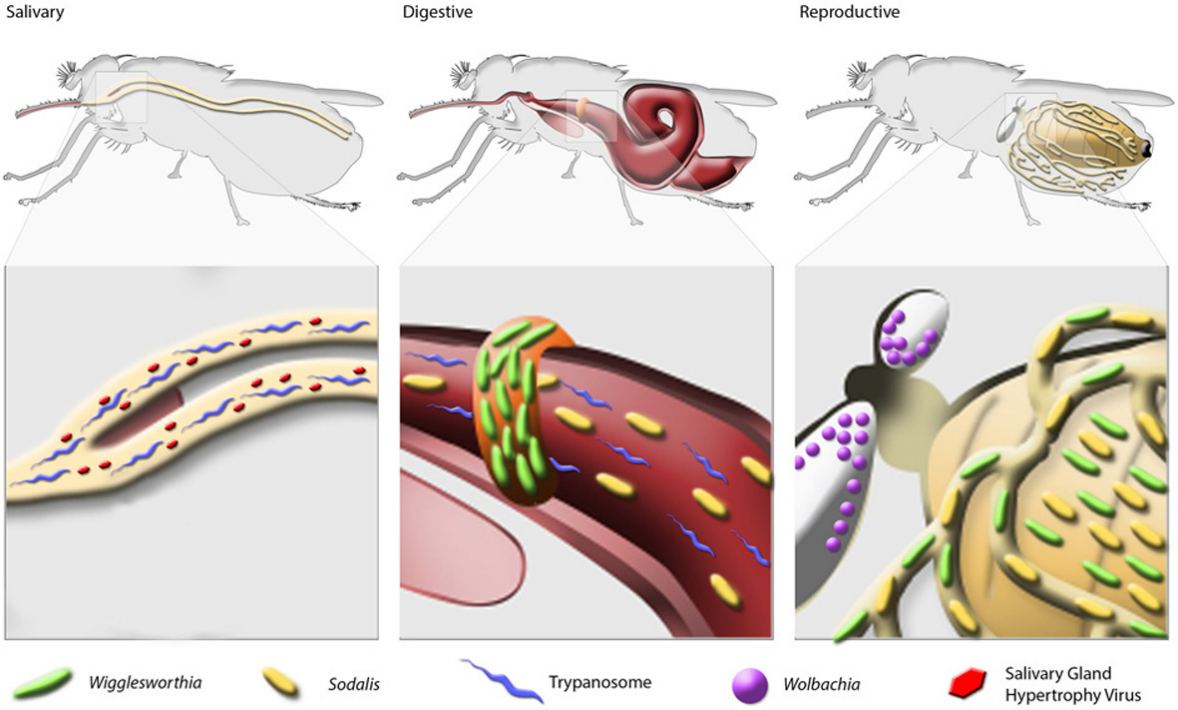
**Localization of symbionts and SGHV in tsetse**.

Bacterial endosymbionts play vital roles in insect metabolic processes, the maintenance of fecundity and immune system development (Douglas, 2011). Tsetse flies house 3 endogenous symbionts, *Wigglesworthia*, *Sodalis* and *Wolbachia*, which also impact the physiology of their host (Figure 1).

### wigglesworthia

Maternal milk is more than a source of nourishment for developing tsetse offspring. This substance also serves as a conduit through which vertically-transmitted symbiotic bacteria colonize developing larvae. All laboratory reared tsetse flies examined to date house up to 3 maternally transmitted symbiotic bacteria. The first of these symbionts is the obligate mutualist *Wigglesworthia*. Tsetse's association with this bacterium began 50-80 million years ago, and since this time *Wigglesworthia* has been subjected to strict vertical transmission within its species-specific tsetse host (Chen et al., 1999). This contiguous association accounts for *Wigglesworthia*'*s* extraordinarily streamlined chromosome, which is about 700 kb in size (Akman et al., 2002; Rio et al., 2012). Comparison of the structural organization and gene content of two distant *Wigglesworthia* species analyzed from *Glossina brevipalpis* and *G. morsitans* revealed overall high synteny between the two genomes, similarly high AT biases (over 80%) and highly conserved coding capacity including the absence of dnaA-based DNA replication mechanisms. However, despite extensive conservation, unique genes were identified between the two symbiont genomes that may result in divergent metabolomes impacting host physiology (Rio et al., 2012). Analysis of the *Wigglesworthia morsitans* specific gene set reveals the presence of a complete shikimate biosynthetic pathway, in which 3-deoxyD-arabino-heptulosonate-7-phosphate (DAHP) can be converted into chorismate. This pathway is degraded in the *Wigglesworthia brevipalpis* genome. Downstream of the chorismate pathway, *W. morsitans'* chromosome encodes *pabA*, *pabB* (aminodeoxychorismate synthase II and I, respectively) and *pabC* (4-amino-4-deoxychorismate lyase). These enzymes catalyze the formation of *p*-aminobenzoate from chorismate–an essential component in folate biosynthesis. The *W. morsitans* genome also contains an *aspC* homolog that can be used following chorismate biosynthesis to produce phenylalanine. Interestingly, African trypanosomes are unable to synthesize phenylalanine and folate, yet their genome encodes transporters capable of salvaging both metabolites from their host environment (Berriman et al., 2005). It remains to be seen whether genomic variations noted in the two *Wigglesworthia* species lead to different biosynthetic capabilities. Additionally, further investigations are required to determine if these metabolic distinctions contribute to the differential trypanosome vector competences exhibited by *G. morsitans* and *G. brevipalpis* flies (Harley and Wilson, 1968; Moloo and Kutuza, 1988).

*Wigglesworthia* provides two well-documented functional benefits to its tsetse host. The first benefit is nutritional, as in the absence of this bacterium, intrauterine larval development is stunted and progeny are aborted (Schlein, 1977; Nogge, 1978; Nogge and Gerresheim, 1982; Pais et al., 2008). Interestingly, *Wigglesworthia*'*s* contracted genome encodes an unusually high number of putative vitamin biosynthesis pathways (Akman et al., 2002; Rio et al., 2012). More than 10% of the retained CDSs are involved in the biosynthesis of cofactors, prosthetic groups and carriers, supporting *Wigglesworthia*'s genetic contributions to *de novo* metabolism of biotin, thiazole, lipoic acid, FAD (riboflavin, B2), folate, pantothenate, thiamine (B1), pyridoxine (B6), and protoheme. This genotypic feature supports the theory that *Wigglesworthia* supplements its tsetse host with nutritious metabolites that are naturally present at low titers in vertebrate blood. Similarly, in mosquitoes, some bacteria, including *Serratia*, *Enterobacter*, and *Asaia*, regulate blood digestion and supplement their host with vitamins (Minard et al., 2013). These observations indicate that hematophagy depends on the presence of gut-associated microbes that facilitate blood meal metabolism.

*Wigglesworthia*'*s* second function in tsetse is immunological. More specifically, when flies undergo intrauterine larval development in the absence of this bacterium they present a severely compromised immune system during adulthood. Under these conditions, *Wigglesworthia*-free tsetse are unusually susceptible to infection with normally non-pathogenic *E. coli* K12 and trypanosomes (Wang et al., 2009; Weiss et al., 2011, 2012, 2013).

Tsetse houses two distinct populations of *Wigglesworthia*. The first resides intracellularly within bacteriocytes, which collectively form an organ called the bacteriome that is found immediately adjacent to tsetse's anterior midgut (Aksoy, 1995, 2000). Tsetse's second population of *Wigglesworthia* is found extracellularly in milk gland secretions (Attardo et al., 2008). Interestingly, *Wigglesworthia*'*s* genome contains genes that encode a complete flagellar structure (Akman et al., 2002). Flagella-associated genes (*fliC*, *motA*) are highly expressed in *Wigglesworthia* that reside extracellularly in tsetse's milk gland, but not in the intracellular bacteriome population (Rio et al., 2012). Expression of a functional flagellum in milk-associated *Wigglesworthia* may facilitate vertical transmission of this bacterium from the pregnant female to the developing intrauterine larvae, where the symbiont colonizes juvenile tissues.

### sodalis

Tsetse's second endosymbiont is the commensal *Sodalis*, which is a gram-negative organism closely related to free-living microbes within the *Enterobacteriacaea*. In addition to tsetse, *Sodalis*-allied bacteria are present in several other insects, including stinkbugs and weevils (Kaiwa et al., 2010; Toju et al., 2010). Unlike *Wigglesworthia*, *Sodalis* exhibits a wide tissue tropism and can be found both intra and extra-cellularly in various tissues including midgut, fat body, milk gland, salivary glands and hemocoel (Cheng and Aksoy, 1999; Balmand et al., 2013). *Sodalis*' 4.2 Mb chromosome is similar in size to those of its free living ancestors. However, *Sodalis*' genome exhibits a low coding capacity and an unusually high number of pseudogenes (over 600 genes) in pathways that it likely no longer requires as a resident tsetse endosymbiont (Toh et al., 2006). Interestingly, *Sodalis'* genome contains features typically associated with pathogenic lifestyles, including 3 type three secretion systems (TTSS). The *Sodalis* TTSSs, which function during tsetse's juvenile developmental stages, share high homology with those from *Salmonella* and *Yersinia*. Interestingly, while structural genes associated with these systems are retained, effector protein genes, the products of which are pathogenic to host cells, are lacking (Toh et al., 2006). *Sodalis* also has retained the capacity to synthesize a complete flagellar structure, which may be important for it to colonize intrauterine tsetse offspring. This bacterium can be cultured in cell-free medium, which further indicates its recent association with tsetse and its intermediate status between free living and obligate intracellular bacteria.

*Sodalis* lacks a clearly defined functional role within its tsetse host. More so, several natural tsetse populations lack this bacterium, suggesting that it presents a truly commensal phenotype within its tsetse host. However, several studies indicate that *Sodalis* may play a role in tsetse's ability to vector pathogenic trypanosomes. *Sodalis* has been reported to increase tsetse's susceptibility to trypanosomes by obstructing the trypanocidal activity of host midgut lectins (Welburn et al., 1993). Specifically, a *G. morsitans* line that harbored a high *Sodalis* infection prevalence exhibited greater susceptibility to trypanosomes than did a *G. austeni* line that harbored a low *Sodalis* infection prevalence (Welburn et al., 1993). Additionally, treatment of tsetse with the antibiotic streptozotocin results in the elimination of *Sodalis* exclusively. While the fecundity of these flies does not change, they become more resistant to trypanosome infections (Dale and Welburn, 2001). In *G. palpalis* populations collected in Cameroon, parasite infection prevalence positively correlated with the presence of *Sodalis* in examined flies (Farikou et al., 2010; Soumana et al., 2013). These results suggest that in contrast to *Wigglesworthia*, which increases tsetse refractoriness to trypanosomes, *Sodalis* appears to favor the establishment of trypanosome infections in tsetse.

### wolbachia

Some tsetse populations also harbor a parasitic bacterium from the genus *Wolbachia*. *Wolbachia* is a wide spread alpha-proteobacteria endosymbiont, infecting approximately 70% insects (Hilgenboecker et al., 2008). This bacterium manipulates the reproductive biology of its host through a variety of mechanisms, including cytoplasmic incompatibility (CI), male killing, feminization and parthenogenesis (Werren et al., 2008). Expression of CI occurs when a *Wolbachia* infected male mates with an uninfected female, causing developmental arrest during embryogenesis. In contrast, *Wolbachia* infected females can mate with uninfected males, or with a male infected with the same *Wolbachia* strain, and produce viable offspring. In tsetse, *Wolbachia* is localized exclusively intracellularly in germ line tissues and can be detected in early oocyte, embryo and larvae (Cheng et al., 2000; Balmand et al., 2013). Unlike *Sodalis* and *Wigglesworthia*, which are transmitted via milk gland secretions, *Wolbachia* is transmitted transovarially via germ line cells. In *G. morsitans* laboratory colonies, *Wolbachia* induces CI when *Wolbachia*-cured females mate with wild-type *Wolbachia*-infected males. Offspring from these crosses perish during early embryogenesis while the reciprocal cross survives and produces viable progeny (Alam et al., 2011).

### Salivary gland hypertrophy virus (SGHV)

Both colony-reared and natural tsetse populations harbor a rod-shaped, enveloped DNA virus called Salivary Gland Hypertrophy Virus (SGHV). In tsetse, SGHV can cause hypertrophy of the salivary glands and gonadal lesions (Jaenson, 1978). SGHV can be vertically transmitted via maternal milk gland secretions or horizontally during the feeding process (Abd-Alla et al., 2011). While the majority of SGHV-infected flies are asymptomatic with no apparent loss of host fitness, flies infected with high virus titers exhibit reduced fecundity and lifespan, and display hypertrophied salivary glands (Sang et al., 1999; Abd-Alla et al., 2011). In field populations, infection prevalence of SGHV varies in different species and locations (Alam et al., 2012; Malele et al., 2013).

### Metabolic interdependence of tsetse's microbiota

Selective elimination of tsetse's microbial partners via treatment with antibiotics revealed that these microorganisms may be metabolically dependent on one another for their survival in the tsetse host (Belda et al., 2010; Snyder et al., 2010; Snyder and Rio, 2013; Wang et al., 2013). For example, offspring from female tsetse that are fed a diet supplemented with ampicillin lack obligate *Wigglesworthia* but still retain *Sodalis* and *Wolbachia* (Pais et al., 2008). In the absence of *Wigglesworthia*, *Sodalis* densities decline and are eventually eliminated from subsequent generations. *Wigglesworthia* was shown to provide thiamine to *Sodalis*, which lacks the ability to synthesize this metabolite but has retained a transporter to scavenge it from the environment (Snyder et al., 2010; Wang et al., 2013). The fate of SGHV in tsetse also depends upon the presence of the fly's endogenous bacterial symbionts. Specifically, virus proliferation and transmission decreased in tsetse that lacked either *Wigglesworthia* or all 3 maternally transmitted microbes (Boucias et al., 2013; Wang et al., 2013). Taken together these findings indicate that nutritional dependencies between tsetse's endogenous symbionts not only influence host fitness, but also regulate microbial density and transmission dynamics of the individual partners.

## Establishment of symbiont infections in the gut

A complete understanding of the mechanisms that allow commensal bacteria to evade their host's gut immune mechanisms and enable them to colonize their host's gut is vital for successful paratransgenic applications. Recent work using the tsetse model system indicates that bacterial surface coat modifications may be intimately involved in determining symbiont infection outcomes. One such molecule, outer membrane protein A (OmpA), regulates *Sodalis'* ability to form biofilms, which are essential for this bacterium to colonize tsetse's gut. A mutant *Sodalis* (*Sodalis*^ΔompA^) line that does not express *ompA* was unable to form biofilms *in vitro*. In contrast, wild-type *Sodalis*, and biofilm-defective *E. coli* (*E. coli*^ΔompA^) genetically modified to express *Sodalis ompA* were able to produce these structures (Maltz et al., 2012). OmpA also influences *Sodalis*' *in vivo* colonization competency. Following *per os* inoculation into tsetse that lack their endogenous microbiome (which serves as an alternative source of OmpA), *Sodalis*^ΔompA^ are unable to colonize the fly gut. In contrast, wild-type cells persist in this niche (Maltz et al., 2012). This finding suggests that biofilm formation is essential for *Sodalis* to be able to colonize the host gut, as these structures may protect the bacteria from the hostile effects of the tsetse's immune system. The identification and functional characterization of additional commensal symbiont colonization factors will be useful for enhancing the efficacy of paratransgenesis in insect disease vectors.

## Tsetse-microbe associations in natural populations

To date, all field-collected tsetse flies examined harbor obligate *Wigglesworthia*. In contrast, *Sodalis* infection prevalence in tsetse field populations varies from 0 to 85% (Maudlin et al., 1990; Farikou et al., 2010; Lindh and Lehane, 2011; Alam et al., 2012). Additionally, multiple *Sodalis* genotypes are present in some field-captured tsetse, suggesting that beyond prevalence *Sodalis* is genetically distant between different tsetse species (Geiger et al., 2005; Farikou et al., 2011). Like *Sodalis*, *Wolbachia* infection prevalence in field-captured tsetse differed significantly between different host species and different populations within the same species (Alam et al., 2012; Doudoumis et al., 2012). Based on standard PCR assays, *Wolbachia* can be detected in *G. m. morsitans, G. m. centralis* and *G. austeni* populations, but not *G. tachinoides*. Additionally, low density *Wolbachia* infections are reported in several species and populations, including *G. fuscipes* and *G. morsitans* subspecies (Alam et al., 2012; Schneider et al., 2013). In field *G. m. morsitans*, *Wolbachia* infection prevalence ranges from 10 to 100% depending on their resident habitats (Doudoumis et al., 2012). Some *G. fuscipes* individuals also harbor infections with multiple *Wolbachia* strains (Symula et al., 2013). For example, *Wolbachia* strains detected in Ugandan *G. fuscipes* belong to two different lineages. Furthermore, unusually high *groEL* sequence diversity was present both within and between individuals. Thus, there appears to be high diversity in infection prevalence, density and the circulating strains associated with different tsetse natural populations. Interestingly, three *Wolbachia* genes, *16S rRNA*, *fbpA* and *wsp*, have inserted into the *G. m. morsitans* host chromosome and the ongoing *G. m morsitans* genome project has revealed that insertions from *Wolbachia* into host chromosome may be more extensive (Doudoumis et al., 2012; S. Aksoy and K. Bourtzis, personal communication).

Tsetse's viviparous mode of reproduction serves as a rigorous barrier between immature developmental stages and the microbe-rich external environment. However, several environmentally acquired bacterial commensals were recently found in the guts of wild tsetse (Geiger et al., 2013). Bacteria belonging to 3 phyla (23 species), the *Firmicutes*, *Proteobacteria* and *Actinobacteria*, were characterized from east African *G. fuscipes* (Lindh and Lehane, 2011). *G. p. palpalis*, *G. pallicera*, *G. nigrofusca*, and *G. caliginea* collected in Cameroon and Angola were found to harbor cultured bacteria from 9 distinct genera (Geiger et al., 2009, 2011). The relative densities of these infections and their persistent association with the tsetse remains to be elucidated. Taxonomically diverse commensal bacteria housed in wild tsetse flies may also influence their host's physiology and vector competence. However, further studies are required to verify this hypothesis.

## Conclusions and future directions

Tsetse flies house several microorganisms, all of which interact with one another and their host to manipulate the physiology of the system as a whole. With the aid of the *Wigglesworthia*, *Sodalis*, *Wolbachia*, SGHV and *T. brucei* sequenced and annotated genomes (the annotation of *G. morsitans* is almost complete), more in depth studies on the interactions between tsetse's multiple partners can be performed. Work to date has focused primarily on host-microbe interactions in laboratory-reared tsetse. However, a growing number of studies using tsetse, and other insect disease vectors, indicate greater spatial and temporal diversity of symbiotic composition, density and prevalence in natural populations. Future work with natural populations will be important to understand the functional role of this diversity on host fitness and pathogen infection outcomes.

### Conflict of interest statement

The authors declare that the research was conducted in the absence of any commercial or financial relationships that could be construed as a potential conflict of interest.
